# Development of DEEP-URO, a Generic Research Tool for Enhancing Antimicrobial Stewardship in a Surgical Specialty

**DOI:** 10.3390/antibiotics15010074

**Published:** 2026-01-09

**Authors:** Eva Falkensammer, Béla Köves, Florian Wagenlehner, José Medina-Polo, Ana-María Tapia-Herrero, Elizabeth Day, Fabian Stangl, Laila Schneidewind, Jennifer Kranz, Truls Erik Bjerklund Johansen, Zafer Tandogdu

**Affiliations:** 1 Department of Urology, Klinikum Wels-Grieskirchen, 4600 Wels, Austria; 2 Department of Pediatric Surgery, Salzburg University Hospital, Paracelsus Medical University, 5020 Salzburg, Austria; 3 Department of Urology, University of Szeged, 6725 Szeged, Hungary; 4 Clinic for Urology, Pediatric Urology and Andrology, Justus-Liebig-University Giessen, 35390 Giessen, Germany; 5 Department of Urology, Hospital Universitario 12 de Octubre, 28041 Madrid, Spain; 6 Complejo Asistencial Universitario de Palencia, University of Valladolid, 47012 Valladolid, Spain; 7 Department of Urology, Ayr University Hospital, Ayr KA6 6DX, UK; 8 Department of Urology, University Hospital of Bern, 3010 Bern, Switzerland; 9 Department of Urology, Salzburg University Hospital, 5020 Salzburg, Austria; 10 Department of Urology and Paediatric Urology, Uniklinik RWTH Aachen, 52074 Aachen, Germany; 11 Department of Urology and Kidney Transplantation, Martin-Luther-University, 06108 Halle, Germany; 12 Department of Urology, Oslo University Hospital, 0315 Oslo, Norway; 13 Institute of Clinical Medicine, University of Oslo, 0315 Oslo, Norway; 14 Department of Urology, University College London Hospitals, London W1G 8PH, UK

**Keywords:** surgical interventions, urology, antibiotic prophylaxis, antimicrobial resistance, postoperative infections, antimicrobial stewardship

## Abstract

**Introduction**: The appropriate use of antibiotic prophylaxis (AP) in surgical procedures is an ongoing debate. There is a lack of evidence, and urological guidelines provide limited, procedure-specific recommendations. Our aim was to develop a generic model of an audit to define the need for AP in urological procedures, as well as in other surgical specialties. **Material and Methods**: Based on our experience with the Global Prevalence of Infections in Urology (GPIU) study and a literature review, we defined benchmark standards for 30-day infection rates, including sepsis, and estimated the number of patients needed to be included in a comparative study of AP versus no AP for a surgical procedure within one year. The generic study model was developed during a modified consensus process within the UTISOLVE research group. Urology departments giving and not giving AP were invited to join our development project as an extension of GPIU. **Results**: Radical prostatectomy was used as a model procedure. Ca. 60 urology centers performing more than 50 radical prostatectomies per year signed up. There was variation in AP practice among sites. Our own review showed that infection rates were ca. 5%, with severe infections, including sepsis, occurring in <0.5% of cases. A sample of 1825 patients would be required to achieve a 95% confidence interval half-width of ±1.0% for general infections. For sepsis, assuming an incidence of 0.5%, a sample of 2124 patients would be needed to reach a 95% confidence interval precision of ±0.30%. Enrollment of 2070 consecutive procedures would be needed to yield precisions of ±0.94% for infection and ±0.30% for sepsis. Based on the number of procedures performed and the number of interested study sites, we agreed on a prospective, multi-center, non-interventional service evaluation, expected to collect standardized data over a 3-month period. The primary outcome was defined as the 30-day incidence of infectious complications. All patients will undergo 30-day post-procedure follow-up through routine clinical care pathways. **Conclusions**: Our audit model is based on benchmarking of relevant outcomes. It defines how to assess AP in surgical procedures and clarifies a series of issues necessary to defend the status of a generic study model. We regard DEEP-URO to be a comprehensive, multi-center-based initiative that will help balance infection prevention with antimicrobial stewardship and improve the quality of clinical practice and personalized medicine.

## 1. Introduction

Antimicrobial resistance (AMR) is one of the most pressing global health challenges of our time, with surgical prophylaxis practices playing a significant contributory role [1]. In urological surgery, the balance between infection prevention and judicious antibiotic use remains poorly defined, even for robot-assisted laparoscopic radical prostatectomy and conventional laparoscopic radical prostatectomy (RA/LP), which are among the most commonly performed operations [2,3]. Infection rates are approximately 5% overall, with severe infection rates below 0.5% [4]. Despite these modest infection rates, antibiotic prophylaxis (AP) practices demonstrate considerable variation across institutions and geographic regions. Up to 45% of patients undergoing urological procedures are exposed to inappropriate antibiotic prophylaxis, either through unnecessary use, incorrect agent selection, or inappropriate duration [5]. We believe the situation is the same for common procedures in other surgical specialties.

The lack of specific guidance in contemporary urology guidelines has perpetuated heterogeneous AP practices [6]. This variation occurs across multiple domains: whether prophylaxis shall be administered, the choice of antibiotic agent, and the duration of prophylaxis. Such inconsistency not only reflects uncertainty in optimal practice but also contributes to the escalating AMR crisis, which in turn negatively impacts outcomes of surgical procedures. The World Health Organization’s AWaRe (Access, Watch, Reserve) classification system provides a structured framework for antibiotic stewardship and recommends that the Access group antibiotics account for at least 60% of total antibiotic consumption [7]. However, implementation of this classification in urological surgical prophylaxis has not been systematically evaluated.

Current literature lacks robust, real-world data examining the relationship between AP variation and infection outcomes. Several critical questions remain unanswered, such as (a) the actual infection risk when AP is withheld in contemporary practice, (b) how the choice of antibiotic agent (particularly when classified by WHO AWaRe categories) impacts infection rates, (c) what is the optimal duration of AP, and (d) which proportion of infections demonstrate resistance to the prophylactic antibiotic used? [4].

Without standardized protocols and comprehensive outcome data, clinical decision-making relies on extrapolation from other surgical contexts, expert opinion, and institutional tradition rather than evidence specific to each procedure. Urologists ran the Global Prevalence study of Infections in Urology (GPIU) for twenty years and studied infection rates, pathogens, and resistance in urology departments, and provided quality assurance data. Unfortunately, our platform and study design did not allow us to perform studies that would allow us to publish high-level evidence to inform guidelines, and especially not to improve AMR. DEEP-URO is a new initiative, and the acronym has a double meaning. The letters stand for DE-Escalation of antibiotic Prophylaxis, but also tell that this time we will dig deeper to seek the truth: *solidum petit in profundis*, which is the motto of one of the participating universities [8].

## 2. Results

### 2.1. Organizational Items

#### 2.1.1. Study Design

DEEP-URO is a generic, prospective, non-interventional, multi-center service evaluation. In principle, the study is a registry-embedded trial. The model is based on observation of routine patient management without deviation from local protocols or introduction of experimental interventions. Each participating site shall include all consecutive eligible patients during a defined 3-month recruitment period, with a 30-day post-operative follow-up for each patient.

#### 2.1.2. Study Setting

The evaluation shall be conducted across multiple surgery centers internationally. To facilitate global collaboration and knowledge sharing, centers from various geographic regions shall be invited to participate, enabling investigation of variations in local infection control protocols and patient populations. High-recruiting centers from the Global Prevalence of Infections in Urology (GPIU) network [5] and other collaborating networks shall be approached.

Study centers shall be invited individually to participate and will be provided with standardized organizational and administrative guidance prior to enrollment. Each center completes a preliminary survey detailing their average number of eligible cases per week for the procedure being studied, local AP protocols, routine follow-up procedures, and internal data management workflows. All eligible consecutive patients undergoing the selected urologic procedure within the agreed 12-week audit period will be included, with a standardized 30-day follow-up. Eligible patients are identified through surgical schedules, with eligibility verified using a standardized checklist to ensure consistent case inclusion across sites.

#### 2.1.3. Site Eligibility Criteria

Participating study centers must meet the following criteria:

- Provide routine follow-up for the patients undergoing the procedure being studied at or around day 30 post-surgery.
- Ability to access and extract data from operative notes (for AP variation documentation) and standard follow-up documentation (clinic letters, discharge summaries, readmission notes).
- Obtain local audit registration/governance approval.
- Demonstrate ability to maintain data completeness and quality standards (≥95%).
- Ability to confirm infection outcomes through local records or coordinated communication with downstream care providers.
- Centers with care pathways that discharge patients without structured postoperative follow-up (e.g., no day 30 contact) shall be excluded.

#### 2.1.4. Patient Eligibility Criteria

The inclusion criterion shall be all consecutive patients undergoing the selected procedure during the 3-month recruitment period. Patients must be >18 years old and have a preoperative urine culture with no microbiological evidence of asymptomatic bacteriuria (ABU).

Exclusion criteria shall be risk factors for surgical field contamination such as indwelling catheters (any type) or intermittent self-catheterization, preoperatively treated urinary tract infection (UTI) within 3 months prior to the operation, preoperatively eradicated ABU, presence of untreated ABU (according to CDC), absence of a preoperative urinary culture or its result, and infective complication following the latest preprocedural biopsy (i.e., prostate biopsy before surgical interventions on the prostate).

In case of operations on the prostate, patients with previous hormonal manipulation, chemotherapy, immunotherapy, any form of focal ablative modalities, or radiotherapy must also be excluded. An eligibility checklist shall be completed by the local audit lead or delegate to confirm fulfillment of entry criteria using the DEEP-URO REDCap screening questionnaire.

Eligible patients can be identified through several mechanisms, such as coordination with waiting list administrators, weekly planning of upcoming operating lists, daily review of operating schedules and theater logbooks, multidisciplinary team (MDT) meeting lists, and clinic appointment records, as well as administrative booking systems.

### 2.2. Outcomes

#### 2.2.1. Outcome Measures and Audit Benchmark Values

The primary indicator is the 30-day infective complication rate, defined as the number of surgical site infections (SSIs), healthcare-associated urinary tract infections (HAUTIs), including catheter-associated UTIs (CAUTIs), bloodstream infections (BSIs), and *Clostridium difficile* infections occurring within 30 days, divided by the number of operations performed. The predefined benchmark standard for 30-day infective complications is 5% [4,9,10].

Secondary outcome measures include the 30-day sepsis rate, defined according to the Sepsis-3 criteria, with a benchmark of 0.5% [4,10]. Further surgical interventions for postoperative complications within 30 days are assessed against a benchmark rate of 1.7% [11]. Hospital readmissions related to infection within 30 days are compared with a benchmark of <1% [9]. The 30-day mortality rate is evaluated against a benchmark standard of 0.001% [12]. An additional exploratory indicator, the AP failure rate, defined as infection with pathogens resistant to the prophylactic antibiotic, shall be assessed using an exploratory benchmark of 25% [13,14].

#### 2.2.2. Case Definitions

*Patient characterization*. In order to prepare for the variability of results due to comorbidities and geographical regions, all patients will be characterized according to the ORENUC system for risk factors for UTI. This includes detailed reporting of patient-related and microbiological findings.

*Infectious complications* are categorized according to the European Centre for Disease Control (ECDC) surveillance protocol and ECDC definitions [15]. Detailed definitions are presented in Appendix S2 (Supplementary Materials). Based on ECDC criteria, infections shall be classified as superficial incisional (SSI-S), deep incisional (SSI-D), organ/space (SSI-O), urinary tract infection (UTI), catheter-associated UTI (CAUTI), or *Clostridium difficile* infection, all confirmed microbiologically.

*Sepsis* is defined according to the Sepsis-III criteria as life-threatening organ dysfunction resulting from dysregulated host response to infection [16], with septic shock captured as a predefined subset (clinical criteria are provided in Appendix S3, Supplementary Materials).

*Further surgical interventions* include procedures such as embolization or drainage of pelvic hematoma, open or laparoscopic intervention for hematoma, urinary diversion for anastomotic complications, refashioning of anastomosis, laparotomy for bowel injury, lymphocele management, fasciotomy for compartment syndrome, and other relevant interventions.

#### 2.2.3. Antibiotic Prophylaxis Assessment

Variation in antibiotic prophylaxis (AP) shall be evaluated across three key domains. First, the presence of AP shall be defined as administration of antibiotics within 0–120 min before the first incision. The absence of AP is defined as no antibiotics administered within 0–120 min before the procedure and none given immediately postoperatively. Second, the duration of AP shall be categorized as “periprocedural”, meaning antibiotics given within 0–120 min before incision, including repeat intraoperative doses in cases of elevated infection risk (e.g., blood loss > 1.5 L or prolonged surgery), or “extended”, defined as any postoperative antibiotic course beyond the periprocedural period. Third, antibiotics shall be classified according to the WHO AWaRe framework [7] into Access (first-/second-choice empiric therapy with lower resistance potential), Watch (agents with higher resistance risk), and Reserve (last-resort antibiotics for multidrug-resistant organisms). The audit benchmark stipulates that at least 60% of antibiotic use should derive from the Access group. (Complete antibiotic categorization is provided in Appendix S1, Supplementary Materials).

#### 2.2.4. Quality of SSI Preventive Measures

Compliance with WHO-recommended SSI preventive measures [2,17] shall be evaluated across participating units. Measures to be assessed include the use of alcohol- and chlorhexidine gluconate-based antiseptic solutions for surgical site skin preparation, adherence to standardized hair-removal protocols, maintenance of perioperative normothermia, and compliance with perioperative blood glucose monitoring and control. Adherence shall be reported in aggregate and classified at the unit level.

#### 2.2.5. Follow-Up and Outcome Assessment

Infections shall be assessed 30 days after the selected procedure through routine follow-up pathways. Accepted surveillance methods include face-to-face clinical review, telephone or postal follow-up, day-30 review (±10 days) by telephone or face-to-face, reports from general practitioners or treating clinicians, and clinically verified patient-reported outcomes. Supplementary methods, such as readmission data review and electronic health record (EHR) screening, may support case identification but are not considered sufficient for confirmation without clinical follow-up, as EHR-only review may fail to capture events outside documented encounters.

Infections arising during the index admission shall be identified through daily case screening by trained site teams and systematic monitoring of clinical and microbiological records. Readmissions within 30 days shall be fully recorded by admitting teams, including clinical and microbiological details.

Post-discharge surveillance relies on routine follow-up pathways and verified patient reports. A structured day-30 review serves as the final surveillance point. After discharge, patients are, of course, free to contact their primary care physician or local hospitals, but they are encouraged to report any need for medical assistance at the 30-day follow-up contact with the study site. For example, in RALP, variations in Trial Without Catheter (TWOC) timing, cystogram use, and TWOC location are also recorded due to their potential influence on infection outcomes.

### 2.3. IT and Statistics

#### 2.3.1. Data Collection and Management

All centers shall enter data into a secure REDCap platform (https://project-redcap.org) currently hosted within the UCL Data Safe Haven. Access shall be restricted to authorized site investigators, and authenticated access is granted to each participating site. All data shall be anonymized at the source, with no patient- or hospital- identifiable information uploaded. Information about participating hospital departments will be publicly available. Information about patient identity will be kept at each hospital, but only anonymous data will be submitted to study centers for population analysis. In case of territorial diversities or patient outliers, both departments and patients may be identified for separate analysis after necessary ethical permissions have been obtained.

#### 2.3.2. Sample Size Calculation

The sample size shall be calculated to estimate primary and secondary indicators with predefined precision. For our first planned study on RA/LP, where the primary indicator is based on an assumption of a 5% 30-day infection rate, a sample of 1825 patients is required to achieve a 95% confidence interval half-width of ±1.0%. For sepsis, assuming an incidence of 0.5%, a sample of 2124 patients was needed to reach a 95% confidence interval precision of ±0.30%. The planned enrollment of 2070 consecutive procedures is expected to yield precisions of ±0.94% for infection and ±0.30% for sepsis, sufficient for audit objectives and accommodating potential clustering across centers. Table S4 in Appendix S5 shows the interrelationship between outcome, assumed incidence, and sample size with 95% CI half-width and enrollment precision for our first planned study.

#### 2.3.3. Statistical Analysis

Continuous variables shall be summarized using means, standard deviations, ranges, and medians, whereas categorical variables shall be presented as counts and percentages. Comparisons of continuous variables employ Student’s *t*-test or the Mann–Whitney test, and categorical variables are compared using the chi-square test or Fisher’s exact test. Only patients with complete follow-up shall be included in the statistical analysis.

Data will be pooled across centers for primary audit outcomes, with site-level results described descriptively. Clustering effects are expected to be minimal due to low event rates. Reporting will be conducted in an aggregated format, with anonymization of surgeons, hospitals, and regions. Local sites have access to summary statistics, while national audit leads have access to country-level data.

#### 2.3.4. Data Quality Standards

Centers achieving <95% data completeness shall receive enhanced oversight and support. Persistent data incompleteness leads to site data exclusion from the final analysis. The audit coordinator shall systematically review completed electronic case report forms (eCRFs) for protocol adherence and missing or inconsistent variables and contact sites when clarification is required.

#### 2.3.5. Quality Assurance and Validation

All participating centers shall receive standardized training through a central online session before audit initiation. Surveillance procedures are aligned with ECDC methodology, and monthly quality-assurance meetings are conducted during data collection. A central audit support team shall be available during working hours via email and Slack messaging.

Random validation shall be conducted at one-third of recruiting sites. Independent validators, nominated in collaboration with national and local audit leads, shall compare eligible patients with reported cases. Sites with minor discrepancies are retained, whereas those with significant inconsistencies undergo detailed review and might be excluded.

### 2.4. Ethics and Governance

#### 2.4.1. Ethical Considerations

A DEEP-URO audit is classified as a service evaluation focusing exclusively on routine patient care, without deviations from established clinical pathways, without collection of identifiable data, and without experimental interventions. According to laws in most countries, this type of audit will be regarded as a quality assurance audit, which does not require ethical approval. For the first planned DEEP-URO study on RALP, the UK Health Research Authority (IRAS Project ID: 344425, 18 August 2025) concluded that formal ethical approval or clinical trials registration was not required. Each participating site remains responsible for meeting local regulatory requirements, securing necessary approvals or audit registration, and complying with institutional policies. Agreements must be signed between each study site and the audit center to ensure safe submission and storage of anonymized patient data.

#### 2.4.2. Data Protection and Confidentiality

Patient confidentiality will be ensured through the use of unique anonymous identifiers, with sites maintaining internal traceability for validation. Hospitals are coded anonymously, preventing identification in audit reports. All collaborators adhere to a strict data-handling code of conduct, with data anonymized at the source and stored in the secure REDCap platform with authenticated access.

#### 2.4.3. Governance Structure

Principal investigators must be defined for each DEEP-URO study. Separate steering groups must be established for each procedure being studied. The steering group works through national coordinators and site investigators. For the first DEEP-URO study, the central audit team at UCL will manage the REDCap system, coordinate training, and oversee data monitoring. National audit leads facilitate site coordination, local approvals, and liaison with national urological societies. Local audit teams, led by designated site investigators, will ensure accurate case capture, timely follow-up, and data submission.

#### 2.4.4. Publication and Authorship

Results shall be reported only in aggregated form and be disseminated through audit reports and peer-reviewed publications, with approval from national audit leads. Authorship shall follow ICMJE guidelines, with national and local leads included as group authors under “DEEP-URO Audit Investigators” and local associates acknowledged.

#### 2.4.5. End of Audit Definition

A DEEP-URO audit concludes when follow-up is completed for the final enrolled patient and when all data have been submitted to the central database. The REDCap platform shall be closed 30 days after the last eligible patient’s 30-day follow-up window.

**Figure 1:**
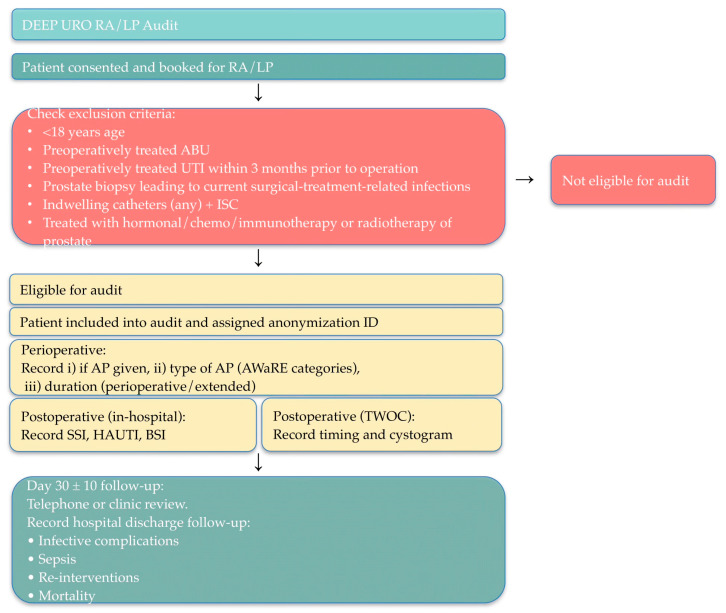
Example of DEEP-URO study pathway. RA/LP = robot-assisted and conventional laparoscopic radical prostatectomy, UTI = urinary tract infection, ABU = asymptomatic bacteriuria, ISC = intermittent self-catheterization, ID = identifier, AP = antibiotic prophylaxis, AWaRE = Access, Watch, and Reserve, SSI = surgical site infection, HAUTI = hospital-acquired urinary tract infection, BSI = bloodstream infection, TWOC = Trial Without Catheter.

Figure 1 shows the study pathway for DEEP-URO RA/LP, which is a multi-center prospective, observational audit of infective complications following robot-assisted or conventional laparoscopic radical prostatectomy. Eligible consecutive patients will be audited after a predefined 12-week period, and each recruited patient will be followed for 30 days. The primary objective is to determine the variation in antibiotic prophylaxis practices and their relationship with 30-day infective complication rates following surgery.

## 3. Discussion

### 3.1. Implications for AMS and Clinical Practice

The DEEP-URO studies will address a critical gap in contemporary urological practice by providing prospectively collected, real-world evidence on the relationship between antibiotic prophylaxis practices and the rates of infectious complications. The multi-center evaluations are expected to give several important contributions to antimicrobial stewardship in urology. By benchmarking current AP practices against established standards (ECDC, WHO AWaRe), the audit will identify opportunities for practice harmonization and highlight variations that may be clinically unjustified. The classification of AP using WHO AWaRe categories will enable assessment of compliance with international stewardship targets and identify opportunities for de-escalation without compromising patient safety. The prospective collection of infection outcomes across varied AP strategies will provide evidence to inform future guideline development and support data-driven decision-making in antibiotic prophylaxis. Study findings will contribute to AMR risk stratification models. Local centers will receive benchmarked data, enabling the identification of areas for improvement and tracking of outcomes over subsequent audit cycles. The generic study model based on the principles of registry-embedded trials and estimations of sample sizes depending on outcomes means that the same model can be used to assess the need for AP in numerous urological procedures, as well as for procedures in other surgical specialties.

We believe the study nature of an audit means that ethical approval is not needed in most hospitals, and recruitment of study centers will be easier. For individual centers, the findings in DEEP-URO studies will represent valuable quality assurance data to inform local AP protocols and guidelines and thereby improve personalized medicine.

### 3.2. Strengths and Weaknesses

The prospective study design of DEEP-URO will reduce bias and improve data quality compared to retrospective reviews. Multi-center international participation will enhance generalizability and enable evaluation of practice variation across healthcare systems. Use of standardized definitions like ECDC and Sepsis-3 definitions will ensure consistency and comparability of results. A comprehensive follow-up of a 30-day surveillance period will capture most of the postoperative infections. The observational design will provide real-world evidence reflecting actual clinical practice without artificial trial constraints. The model has multiple mechanisms ensuring data quality and completeness.

A weakness of our generic model is the risk of selection bias. To counteract this limitation and preserve generalizability, the model will exclude centers without structured follow-up. There is a risk that an observational design will limit establishments of causality and imply residual confounding. Heterogeneity due to variation in local practices may complicate interpretation. Variability in surveillance methods (telephone vs. face-to-face) may affect outcome detection. Despite quality standards, some data incompleteness must be expected. Finally, geographic variation in AMR may limit the generalizability of findings.

### 3.3. Further Research

Following completion of a DEEP-URO study on a given procedure, several follow-up activities can be foreseen. Repeat audit cycles will enable assessment of practice changes and outcome trends over time. Findings may generate hypotheses for future interventional studies and inform the design of randomized controlled trials testing specific AP strategies and facilitate the development of evidence-based, individualized antibiotic prophylaxis strategies tailored to patient-specific risk factors and procedural characteristics. DEEP-URO may evaluate the feasibility of multi-center infection surveillance. In general, study findings will help identify knowledge gaps requiring further investigation.

Once implemented, the DEEP-URO model can easily be extended to other urological procedures. Our first planned audit will address radical prostatectomy for prostate cancer, and the second will address transurethral resection of benign prostates causing obstruction. Accumulated results can be used for the development of general risk prediction models for postoperative infection. Future studies should include health economics and cost-effectiveness analysis of different AP strategies.

## 4. Methods

Our primary objective was to design a generic model of an audit or a study that could demonstrate the 30-day healthcare-associated surgical infection rates across variations in antibiotic prophylaxis (use vs. no use, type of agent, duration) in patients undergoing specific urological procedures.

Our secondary objectives were composite (a) to be able to assess the 30-day rate of sepsis (Sepsis-3 definition) [16] (b) determine the frequency of additional surgical interventions within 30 days of surgery, (c) evaluate AP practices against WHO AWaRe classification benchmarks [7], (d) assess the rate of AP failure in microbiologically confirmed infections, (e) determine 30-day mortality rates, (f) evaluate hospital readmission rates for infective complications, and (g) assess compliance with established surgical site infection (SSI) preventive measures [17].

Our developmental work was a continuous consensus process during several years at international congresses, at regular monthly online meetings, and through extensive communication by e-mail. The model has also been presented and discussed in the *European Urology Today*, which was the official newsletter of the European Association of Urology (EAU) [18]. During the development process, UTISOLVE [19] was established as the executive research group of the European Section of Infection in Urology, which is a full scientific section under EAU. The group is seeking affiliation with a university where one of the senior group members holds an academic position. The results of our development process are a detailed description of core aspects of our generic study model.

## 5. Conclusions

DEEP-URO represents a comprehensive, multi-center initiative to address the critical challenge of balancing infection prevention with antimicrobial stewardship in urological procedures. The generic study design can easily be adopted in other surgical specialties. By systematically evaluating real-world antibiotic prophylaxis practices and their relationship with 30-day infection outcomes, this audit model will generate essential evidence to guide clinical practice, inform guidelines, and support quality improvement efforts in the context of the global antimicrobial resistance crisis.

The standardized methodology, international collaboration, and rigorous quality assurance mechanisms position DEEP-URO to provide robust, generalizable findings that will improve personalized medicine and contribute meaningfully to the ongoing efforts to optimize antibiotic use while maintaining patient safety in urological surgery.

## Data Availability

The protocol and data collection forms of the DEEP-URO audits will be available from the corresponding author upon reasonable request. Individual patient data will not be made publicly available to protect patient privacy. Aggregated, anonymized results will be published in peer-reviewed journals following study completion.

## Supplementary Materials

The following supporting information can be downloaded at: https://www.mdpi.com/article/10.3390/antibiotics15010074/s1, Appendix S1: WHO AWaRe Antibiotic Classification; Appendix S2: ECDC Surgical Site Infection Definitions; Appendix S3: Sepsis-3 Clinical Criteria; Appendix S4: Data Collection Forms (these forms exist for DEEP-URO RA/LP and are available upon request); Hospital Form Data Library; Patient Screening Form; Patient Data Collection Table. Appendix S5: Sample Size Calculation. Refs. [4,20,21,22] are cited in the Supplementary Materials.

## Abbreviations

AMR	Antimicrobial resistance
AP	Antibiotic prophylaxis
ABU	Asymptomatic bacteriuria
AWaRe	Access, Watch, Reserve (WHO classification)
BSI	Bloodstream infection
CAUTI	Catheter-Associated Urinary Tract Infection
CHG	Chlorhexidine gluconate
CPIC	Clinical Pharmacogenetics Implementation Consortium
DEEP-URO	De-escalation of AP in Urological Procedures
EAU	European Association of Urology
ECDC	European Centre for Disease Prevention and Control
eCRF	Electronic case report form
EHR	Electronic health record
GPIU	Global Prevalence of Infections in Urology
HAI	Healthcare-associated infection
HAUTI	Healthcare-associated urinary tract infection
ICMJE	International Committee of Medical Journal Editors
IPC	Infection Prevention and Control
LP	Laparoscopic Prostatectomy
MDT	Multidisciplinary team
NAL	National audit lead
RALP	Robot-assisted laparoscopic radical prostatectomy
RA/LP	Robot-Assisted/Laparoscopic Prostatectomy
REDCap	Research Electronic Data Capture
SSI	Surgical site infection
TWOC	Trial Without Catheter
UCL	University College London
UTI	Urinary tract infection
WHO	World Health Organization

## References

[1] de la santé O.M. (2014). Antimicrobial Resistance: Global Report on Surveillance.

[2] Allegranzi B., Bischoff P., De Jonge S., Kubilay N.Z., Zayed B., Gomes S.M., Abbas M., Atema J.J., Gans S., Van Rijen M. (2016). New WHO Recommendations on Preoperative Measures for Surgical Site Infection Prevention: An Evidence-Based Global Perspective. Lancet Infect. Dis..

[3] Bootsma A.M.J., Laguna Pes M.P., Geerlings S.E., Goossens A. (2008). Antibiotic Prophylaxis in Urologic Procedures: A Systematic Review. Eur. Urol..

[4] Falkensammer E., Erenler E., Johansen T.E.B., Tzelves L., Schneidewind L., Yuan Y., Cai T., Koves B., Tandogdu Z. (2023). Antimicrobial Prophylaxis in Robot-Assisted Laparoscopic Radical Prostatectomy: A Systematic Review. Antibiotics.

[5] Çek M., Tandoğdu Z., Naber K., Tenke P., Wagenlehner F., Van Oostrum E., Kristensen B., Bjerklund Johansen T.E. (2013). Antibiotic Prophylaxis in Urology Departments, 2005–2010. Eur. Urol..

[6] https://uroweb.org/guidelines/urological-infections.

[7] The Selection and Use of Essential Medicines, 2025: WHO AWaRe (Access, Watch, Reserve) Classification of Antibiotics for Evaluation and Monitoring of Use. https://www.who.int/publications/i/item/B09489.

[8] The History of Aarhus University in 25 Sections/The University’s Seal. https://auhist.au.dk/en/25kapitlerafuniversitetetshistorie/theuniversity%27sseal.

[9] Moschini M., Gandaglia G., Fossati N., Dell’Oglio P., Cucchiara V., Luzzago S., Zaffuto E., Suardi N., Damiano R., Shariat S.F. (2017). Incidence and Predictors of 30-Day Readmission After Robot-Assisted Radical Prostatectomy. Clin. Genitourin. Cancer.

[10] Merhe A., Abou Heidar N., Hout M., Bustros G., Mailhac A., Tamim H., Wazzan W., Bulbul M., Nasr R. (2020). An Evaluation of the Timing of Surgical Complications Following Radical Prostatectomy: Data from the American College of Surgeons National Surgical Quality Improvement Program (ACS-NSQIP). Arab. J. Urol..

[11] Wallerstedt Lantz A., Stranne J., Tyritzis S.I., Bock D., Wallin D., Nilsson H., Carlsson S., Thorsteinsdottir T., Gustafsson O., Hugosson J. (2019). 90-Day Readmission after Radical Prostatectomy-a Prospective Comparison between Robot-Assisted and Open Surgery. Scand. J. Urol..

[12] Björklund J., Stattin P., Rönmark E., Aly M., Akre O. (2022). The 90-Day Cause-Specific Mortality after Radical Prostatectomy: A Nationwide Population-Based Study. BJU Int..

[13] Tandoğdu Z., Bartoletti R., Cai T., Çek M., Grabe M., Kulchavenya E., Köves B., Menon V., Naber K., Perepanova T. (2016). Antimicrobial Resistance in Urosepsis: Outcomes from the Multinational, Multicenter Global Prevalence of Infections in Urology (GPIU) Study 2003–2013. World J. Urol..

[14] Daneman N., Low D.E., McGeer A., Green K.A., Fisman D.N. (2008). At the Threshold: Defining Clinically Meaningful Resistance Thresholds for Antibiotic Choice in Community-Acquired Pneumonia. Clin. Infect. Dis..

[15] European Centre for Disease Prevention and Control (2016). Point Prevalence Survey of Healthcare-Associated Infections and Antimicrobial Use in European Acute Care Hospitals: Protocol Version 5.3: ECDC PPS 2016–2017.

[16] Singer M., Deutschman C.S., Seymour C.W., Shankar-Hari M., Annane D., Bauer M., Bellomo R., Bernard G.R., Chiche J.-D., Coopersmith C.M. (2016). The Third International Consensus Definitions for Sepsis and Septic Shock (Sepsis-3). J. Am. Med. Assoc..

[17] Mangram A.J., Horan T.C., Pearson M.L., Silver L.C., Jarvis W.R. (1999). Guideline for Prevention of Surgical Site Infection, 1999. Centers for Disease Control and Prevention (CDC) Hospital Infection Control Practices Advisory Committee. Am. J. Infect. Control.

[18] European Urology Today: April/May 2023. https://online.flippingbook.com/view/257304614?sharedOn=.

[19] UTISOLVE. https://www.linkedin.com/company/utisolveresearch/posts/?feedView=all.

[20] Williams B. (2022). The National Early Warning Score: From Concept to NHS Implementation. Clin. Med..

[21] Levy M.M., Fink M.P., Marshall J.C., Abraham E., Angus D., Cook D., Cohen J., Opal S.M., Vincent J.-L., Ramsay G. (2003). 2001 SCCM/ESICM/ACCP/ATS/SIS International Sepsis Definitions Conference. Intensive Care Med..

[22] Recommendations|Sepsis: Recognition, Diagnosis and Early Management|Guidance|NICE. https://www.nice.org.uk/guidance/NG51/chapter/Recommendations#identifying-people-with-suspected-sepsis.

