# Supplementary material for: Development of DEEP-URO, a Generic Research Tool for Enhancing Antimicrobial Stewardship in a Surgical Specialty
Source: Antibiotics (Basel). 2026 Jan 9;15(1):74. doi: 10.3390/antibiotics15010074 (PMC12837644; doi:10.3390/antibiotics15010074)
Supplement: Supplementary file 1 [file antibiotics-15-00074-s001.zip › antibiotics-4045906-supplementary.pdf]

Development of DEEP-URO, a Generic Research Tool for Enhancing Antimicrobial Stewardship in a Surgical Specialty

Eva Falkensammer [1,2,*], Béla Köves [3], Florian Wagenlehner [4], José Medina-Polo [5], Ana-María Tapia-Herrero [6], Elizabeth Day [7], Fabian Stangl [8,9], Laila Schneidewind [8], Jennifer Kranz [10,11], Truls Erik Bjerklund Johansen [12,13,*,†] and Zafer Tandogdu [14,†] on behalf of the UTISOLVE Research Group

[1] Department of Urology, Klinikum Wels-Grieskirchen, 4600 Wels, Austria
[2] Department of Pediatric Surgery, Salzburg University Hospital, Paracelsus Medical University, 5020 Salzburg, Austria
[3] Department of Urology, University of Szeged, 6725 Szeged, Hungary
[4] Clinic for Urology, Pediatric Urology and Andrology, Justus-Liebig-University Giessen, 35390 Giessen, Germany
[5] Department of Urology, Hospital Universitario 12 de Octubre, 28041 Madrid, Spain
[6] Complejo Asistencial Universitario de Palencia, University of Valladolid, 47012 Valladolid, Spain
[7] Department of Urology, Ayr University Hospital, Ayr KA6 6DX, UK
[8] Department of Urology, University Hospital of Bern, 3010 Bern, Switzerland
[9] Department of Urology, Salzburg University Hospital, 5020 Salzburg, Austria
[10] Department of Urology and Paediatric Urology, Uniklinik RWTH Aachen, 52074 Aachen, Germany
[11] Department of Urology and Kidney Transplantation, Martin-Luther-University, 06108 Halle, Germany
[12] Department of Urology, Oslo University Hospital, 0315 Oslo, Norway
[13] Institute of Clinical Medicine, University of Oslo, 0315 Oslo, Norway
[14] Department of Urology, University College London Hospitals, London W1G 8PH, UK
* Correspondence: e.falkensammer@gmail.com (E.F.); t.e.b.johansen@medisin.uio.no (T.E.B.J.)
† These authors contributed equally to this work.

:

Supplementary Materials

- **Appendix S1:** WHO AWaRe Antibiotic Classification
- **Appendix S2:** ECDC Surgical Site Infection Definitions
- **Appendix S3:** Sepsis-3 Clinical Criteria
- **Appendix S4:** Data Collection Forms (these forms exist for DEEP-URO RA/LP and are available upon request)
    - **Hospital form data library**
    - **Patient screening form**
    - **Patient Data collection table**
- **Appendix S5:** Sample Size Calculation

Supplementary Material

Appendix S1 – WHO AWaRe Antibiotics Classes

AWaRe is a classification system for antibiotics established by the World Health Organization. This classification divides antibiotics into three categories:

1. Access antibiotics: spectrum of activity and a favourable safety profile in terms of side effects.

2. Watch antibiotics: broader-spectrum antibiotics that are recommended as the first-choice treatment for patients with more severe clinical conditions or for infections where the causative pathogens are more likely to be resistant to Access antibiotics.

3. Reserve antibiotics: antibiotics of last resort, reserved for the treatment of multidrug-resistant infections. They are used when other treatment options have been exhausted.

Antibiotic	Class
**Access Group Antibiotics**	
Spectinomycin	Aminocyclitols
Amikacin	Aminoglycosides
Gentamicin	Aminoglycosides
Chloramphenicol	Amphenicols
Thiamphenicol	Amphenicols
Amoxicillin/clavulanic-acid	Beta-lactam/beta-lactamase-inhibitor
Ampicillin/sulbactam	Beta-lactam/beta-lactamase-inhibitor
Sultamicillin	Beta-lactam/beta-lactamase-inhibitor
Sulbactam	Beta-lactamase-inhibitors
Cefacetrile	First-generation-cephalosporins
Cefadroxil	First-generation-cephalosporins
Cefalexin	First-generation-cephalosporins
Cefaloridine	First-generation-cephalosporins
Cefalotin	First-generation-cephalosporins
Cefapirin	First-generation-cephalosporins
Cefatrizine	First-generation-cephalosporins
Cefazedone	First-generation-cephalosporins
Cefazolin	First-generation-cephalosporins
Cefradine	First-generation-cephalosporins
Cefroxadine	First-generation-cephalosporins
Ceftezole	First-generation-cephalosporins
Metronidazole_IV	Imidazoles
Metronidazole_oral	Imidazoles
Ornidazole_IV	Imidazoles
Ornidazole_oral	Imidazoles
Secnidazole	Imidazoles
Tinidazole_IV	Imidazoles
Tinidazole_oral	Imidazoles

Clindamycin	Lincosamides
Furazidin	Nitrofuran derivatives
Nifurtoinol	Nitrofuran derivatives
Nitrofurantoin	Nitrofuran-derivatives
Amoxicillin	Penicillins
Ampicillin	Penicillins
Benzathine-benzylpenicillin	Penicillins
Benzylpenicillin	Penicillins
Cloxacillin	Penicillins
Phenoxymethylpenicillin	Penicillins
Procaine-benzylpenicillin	Penicillins
Azidocillin	Penicillins
Bacampicillin	Penicillins
Clometocillin	Penicillins
Dicloxacillin	Penicillins
Epicillin	Penicillins
Flucloxacillin	Penicillins
Hetacillin	Penicillins
Mecillinam	Penicillins
Metampicillin	Penicillins
Meticillin	Penicillins
Nafcillin	Penicillins
Oxacillin	Penicillins
Penamecillin	Penicillins
Pivampicillin	Penicillins
Pivmecillinam	Penicillins
Propicillin	Penicillins
Talampicillin	Penicillins
Sulfadiazine/tetroxoprim	Sulfonamide-trimethoprim-combinations
Sulfadiazine/trimethoprim	Sulfonamide-trimethoprim-combinations
Sulfadimidine/trimethoprim	Sulfonamide-trimethoprim-combinations
Sulfamerazine/trimethoprim	Sulfonamide-trimethoprim-combinations
Sulfamethoxazole/trimethoprim	Sulfonamide-trimethoprim-combinations
Sulfametrole/trimethoprim	Sulfonamide-trimethoprim-combinations
Sulfamoxole/trimethoprim	Sulfonamide-trimethoprim-combinations
Sulfadiazine	Sulfonamides
Sulfadimethoxine	Sulfonamides
Sulfadimidine	Sulfonamides
Sulfaisodimidine	Sulfonamides
Sulfalene	Sulfonamides
Sulfamazone	Sulfonamides
Sulfamerazine	Sulfonamides
Sulfamethizole	Sulfonamides

Sulfamethoxazole	Sulfonamides
Sulfamethoxypyridazine	Sulfonamides
Sulfametomidine	Sulfonamides
Sulfametoxydiazine	Sulfonamides
Sulfamoxole	Sulfonamides
Sulfanilamide	Sulfonamides
Sulfaperin	Sulfonamides
Sulfaphenazole	Sulfonamides
Sulfapyridine	Sulfonamides
Sulfathiazole	Sulfonamides
Sulfathiourea	Sulfonamides
Sulfafurazole	Sulfonamides
Doxycycline	Tetracyclines
Tetracycline	Tetracyclines
Brodimoprim	Trimethoprim-derivatives
Trimethoprim	Trimethoprim-derivatives
**Reserve**	
Plazomicin	Aminoglycosides
Imipenem/cilastatin/rele-bactam	Carbapenems
Meropenem/vaborbactam	Carbapenems
Ceftaroline-fosamil	Fifth-generation cephalosporins
Ceftobiprole-medocaril	Fifth-generation cephalosporins
Ceftolozane/tazobactam	Fifth-generation cephalosporins
Dalbavancin	Glycopeptides
Oritavancin	Glycopeptides
Telavancin	Glycopeptides
Tigecycline	Glycylcyclines
Daptomycin	Lipopeptides
Aztreonam	Monobactams
Carumonam	Monobactams
Cefiderocol	Other-cephalosporins
Linezolid	Oxazolidinones
Tedizolid	Oxazolidinones
Faropenem	Penems
Fosfomycin_IV	Phosphonics
Lefamulin	Pleuromutilin
Colistin_IV	Polymyxins
Colistin_oral	Polymyxins
Polymyxin-B_IV	Polymyxins
Polymyxin-B_oral	Polymyxins
Dalfopristin/quinupristin	Streptogramins
Eravacycline	Tetracyclines

Minocycline_IV	Tetracyclines
Omadacycline	Tetracyclines
Ceftazidime/avibactam	Third-generation-cephalosporins
Iclaprim	Trimethoprim-derivatives
**Watch**	
Arbekacin	Aminoglycosides
Bekanamycin	Aminoglycosides
Dibekacin	Aminoglycosides
Isepamicin	Aminoglycosides
Kanamycin_IV	Aminoglycosides
Kanamycin_oral	Aminoglycosides
Micronomicin	Aminoglycosides
Neomycin_IV	Aminoglycosides
Neomycin_oral	Aminoglycosides
Netilmicin	Aminoglycosides
Ribostamycin	Aminoglycosides
Sisomicin	Aminoglycosides
Streptoduocin	Aminoglycosides
Streptomycin_IV	Aminoglycosides
Streptomycin_oral	Aminoglycosides
Tobramycin	Aminoglycosides
Piperacillin/tazobactam	Beta-lactam/beta-lactamase-inhibitor_anti-pseudomonal
Tazobactam	Beta-lactamase-inhibitors
Biapenem	Carbapenems
Doripenem	Carbapenems
Ertapenem	Carbapenems
Imipenem/cilastatin	Carbapenems
Meropenem	Carbapenems
Panipenem	Carbapenems
Tebipenem	Carbapenems
Ciprofloxacin	Fluoroquinolones
Delafloxacin	Fluoroquinolones
Enoxacin	Fluoroquinolones
Fleroxacin	Fluoroquinolones
Garenoxacin	Fluoroquinolones
Gatifloxacin	Fluoroquinolones
Gemifloxacin	Fluoroquinolones
Grepafloxacin	Fluoroquinolones
Lascufloxacin	Fluoroquinolones
Levofloxacin	Fluoroquinolones
Levonadifloxacin	Fluoroquinolones
Lomefloxacin	Fluoroquinolones

Moxifloxacin	Fluoroquinolones
Norfloxacin	Fluoroquinolones
Ofloxacin	Fluoroquinolones
Pazufloxacin	Fluoroquinolones
Pefloxacin	Fluoroquinolones
Prulifloxacin	Fluoroquinolones
Rufloxacin	Fluoroquinolones
Sitafloxacin	Fluoroquinolones
Sparfloxacin	Fluoroquinolones
Temafloxacin	Fluoroquinolones
Tosufloxacin	Fluoroquinolones
Trovafloxacin	Fluoroquinolones
Cefepime	Fourth-generation-cephalosporins
Cefoselis	Fourth-generation-cephalosporins
Cefozopran	Fourth-generation-cephalosporins
Cefpirome	Fourth-generation-cephalosporins
Teicoplanin	Glycopeptides
Vancomycin_IV	Glycopeptides
Vancomycin_oral	Glycopeptides
Lincomycin	Lincosamides
Azithromycin	Macrolides
Clarithromycin	Macrolides
Dirithromycin	Macrolides
Erythromycin	Macrolides
Fidaxomicin	Macrolides
Flurithromycin	Macrolides
Josamycin	Macrolides
Midecamycin	Macrolides
Miocamycin	Macrolides
Oleandomycin	Macrolides
Rokitamycin	Macrolides
Roxithromycin	Macrolides
Solithromycin	Macrolides
Spiramycin	Macrolides
Telithromycin	Macrolides
Troleandomycin	Macrolides
Carbenicillin	Penicillins
Carindacillin	Penicillins
Temocillin	Penicillins
Ticarcillin	Penicillins
Aspoxicillin	Penicillins
Azlocillin	Penicillins
Mezlocillin	Penicillins
Pheneticillin	Penicillins

Piperacillin	Penicillins
Sulbenicillin	Penicillins
Clofoctol	Phenol derivatives
Cinoxacin	Quinolones
Flumequine	Quinolones
Nemonoxacin	Quinolones
Oxolinic-acid	Quinolones
Pipemidic-acid	Quinolones
Piromidic-acid	Quinolones
Rosoxacin	Quinolones
Rifabutin	Rifamycins
Rifampicin	Rifamycins
Rifamycin_IV	Rifamycins
Rifamycin_oral	Rifamycins
Rifaximin	Rifamycins
Cefaclor	Second-generation-cephalosporins
Cefamandole	Second-generation-cephalosporins
Cefbuperazone	Second-generation-cephalosporins
Cefmetazole	Second-generation-cephalosporins
Cefminox	Second-generation-cephalosporins
Cefonicid	Second-generation-cephalosporins
Ceforanide	Second-generation-cephalosporins
Cefotetan	Second-generation-cephalosporins
Cefotiam	Second-generation-cephalosporins
Cefoxitin	Second-generation-cephalosporins
Cefprozil	Second-generation-cephalosporins
Cefuroxime	Second-generation-cephalosporins
Flomoxef	Second-generation-cephalosporins
Loracarbef	Second-generation-cephalosporins
Fusidic-acid	Steroid antibacterials
Pristinamycin	Streptogramins
Chlortetracycline	Tetracyclines
Clomocycline	Tetracyclines
Demeclocycline	Tetracyclines
Lymecycline	Tetracyclines
Metacycline	Tetracyclines
Oxytetracycline	Tetracyclines
Penimepicycline	Tetracyclines
Rolitetracycline	Tetracyclines
Sarecycline	Tetracyclines
Cefcapene-pivoxil	Third-generation-cephalosporins
Cefdinir	Third-generation-cephalosporins
Cefditoren-pivoxil	Third-generation-cephalosporins
Cefetamet-pivoxil	Third-generation-cephalosporins

Cefixime	Third-generation-cephalosporins
Cefmenoxime	Third-generation-cephalosporins
Cefodizime	Third-generation-cephalosporins
Cefoperazone	Third-generation-cephalosporins
Cefotaxime	Third-generation-cephalosporins
Cefpiramide	Third-generation-cephalosporins
Cefpodoxime-proxetil	Third-generation-cephalosporins
Cefsulodin	Third-generation-cephalosporins
Ceftazidime	Third-generation-cephalosporins
Cefteram-pivoxil	Third-generation-cephalosporins
Ceftibuten	Third-generation-cephalosporins
Ceftizoxime	Third-generation-cephalosporins
Ceftriaxone	Third-generation-cephalosporins
Latamoxef	Third-generation-cephalosporins
Fosfomycin_oral	Phosphonics
Minocycline_oral	Tetracyclines

Appendix S2 – ECDC Surgical Site Infection Definitions (Table S1)

- Surgical site infections (ECDC –2018)

Note: All definitions are to be assumed to be confirmed for the purposes of surveillance reporting.

Superficial incisional (SSI-S)
Infection occurs within 30 days after the operation AND infection involves only skin and subcutaneous tissue of the incision AND at least one of the following:
1. Purulent drainage with or without laboratory confirmation, from the superficial incision
2. Organisms isolated from an aseptically obtained culture of fluid or tissue from the superficial incision
3. At least one of the following signs or symptoms of infection: pain or tenderness, localized swelling, redness, or heat AND superficial incision is deliberately opened by surgeon, unless incision is culture-negative
4. Diagnosis of superficial incisional SSI made by a surgeon or attending physician

Deep incisional (SSI-D)
Infection occurs within 30 days after the operation if no implant is left in place or within 90 days if implant is in place AND the infection appears to be related to the operation AND infection involves deep soft tissue (for example, fascia, muscle) of the incision AND at least one of the following:

5. Purulent drainage from the deep incision but not from the organ/space component of the surgical site
6. A deep incision spontaneously dehisces or is deliberately opened by a surgeon when the patient has at least one of the following signs or symptoms: fever (> 38 °C), localized pain or tenderness, unless incision is culture-negative
7. An abscess or other evidence of infection involving the deep incision is found on direct examination, during reoperation, or by histopathologic or radiologic examination
8. Diagnosis of deep incisional SSI made by a surgeon or attending physician

Organ/Space (SSI-O)
Infection occurs within 30 days after the operation if no implant is left in place or within 90 days if implant is in place AND the infection appears to be related to the operation AND infection involves any part of the anatomy (for example, organs and spaces) other than the incision which was opened or manipulated during an operation AND at least one of the following:
9. Purulent drainage from a drain that is placed through a stab wound into the organ/space
10. Organisms isolated from an aseptically obtained culture of fluid or tissue in the organ/space
11. An abscess or other evidence of infection involving the organ/space that is found on direct examination, during reoperation, or by histopathologic or radiologic examination
12. Diagnosis of organ/space SSI made by a surgeon or attending physician

- UTI: Urinary tract infection (ECDC-2018)

UTI-A: microbiologically confirmed symptomatic UTI
Patient has at least <u>one</u> of the following signs or symptoms with no other recognized cause: fever (> 38 °C), urgency, frequency, dysuria, or suprapubic tenderness
AND
patient has a positive urine culture, that is, ≥ 105 microorganisms per ml of urine with no more than two species of microorganisms.

UTI-B: not microbiologically confirmed symptomatic UTI
Patient has at least <u>two</u> of the following with no other recognized cause: fever (> 38 °C), urgency, frequency, dysuria, or suprapubic tenderness
AND

at least one of the following:
1. Positive dipstick for leukocyte esterase and/or nitrate
2. Pyuria urine specimen with ≥ 104 WBC/ml or ≥ 3 WBC/high-power field of unspun urine
3. Organisms seen on Gram stain of unspun urine
4. At least two urine cultures with repeated isolation of the same uropathogen (Gram-negative bacteria or *Staphylococcus saprophyticus*) with ≥ 102 colonies/ml urine in nonvoided specimens
5. ≤ 105 colonies/ml of a single uropathogen (Gram-negative bacteria or *Staphylococcus saprophyticus*) in a patient being treated with effective antimicrobial agent for a urinary infection
6. Physician diagnosis of a urinary tract infection
7. Physician institutes appropriate therapy for a urinary infection
Asymptomatic bacteriuria should not be reported, but bloodstream infections secondary to asymptomatic bacteriuria are reported as BSI with source (origin) S-UTI

CAUTI: Catheter associated urinary tract infection
A urinary tract infection is defined as catheter-associated if an indwelling urinary catheter was present (even intermittently) in the 7 days preceding the onset of infection

- Nosocomial infections (ECDC –2018)

GENERAL CASE DEFINITION OF NOSOCOMIAL INFECTION (OR ‘HEALTHCARE-ASSOCIATED INFECTION (HAI)’)
A nosocomial infection associated to the current hospital stay is defined as infection that matches one of the case definitions AND
1. the onset of symptoms was on day 3 or later (day of admission = day 1) of the current hospital admission OR
2. the patient underwent surgery on day 1 or day 2 and develops symptoms of a Surgical Site Infection before day 3 OR
3. an invasive device was placed on day 1 or day 2 resulting in an HAI before day 3
A nosocomial infection associated to a previous hospital stay is defined as an infection that matches one of the case definitions
AND
4. the patient presents with an infection but has been readmitted less than 48 hours after a previous admission to an acute care hospital

OR
5. the patient has been admitted with an infection that meets the case definition of a Surgical Site Infection i.e. the SSI occurred within 30 days of the operation (or in the case of surgery involving an implant was a deep or organ/space SSI that developed within 90 days of the operation) and the patient either has symptoms that meet the case definition and/or is on antimicrobial treatment for that infection
OR
6. the patient has been admitted (or develops symptoms within 2 days) with *Clostridium difficile* infection less than 28 days from a previous discharge from an acute care hospital.
Note: For the purpose of point prevalence surveys, an active nosocomial infection present on the day of the survey is defined as an infection for which signs and symptoms of the infection are present on the survey date or signs and symptoms were present in the past and the patient is (still) receiving treatment for that infection on the survey date. The presence of symptoms and signs should be verified until the start of the treatment in order to determine whether the treated infection matches one of the case definitions of nosocomial infection

Appendix S3 – Sepsis-3 Clinical Criteria

Urinary tract infections can be categorized based on their symptoms, systemic effects, and their association with sepsis.

Diagnosing sepsis does not rely on a single test, and it's complicated by the fact that different regions employ varying tools for sepsis screening. Two well-known tools include the SIRS criteria and the NEWS2 score. While the DEEP-URO RA/LP study aims to identify severe infections and diagnose sepsis, it does not attempt to standardize sepsis screening methods. Therefore, the study will accept the use of either SIRS or NEWS2 screening tools [20,21]. These tools indicate acute deterioration but are not confirmation of sepsis. To confirm a diagnosis of sepsis, the Sepsis-3 definitions will be used [4].
For participating centers in the UK, the NICE 2016 ng51 guidelines on sepsis will be followed for sepsis screening and diagnosis[22]. These guidelines employ a risk stratification approach, and the specific guidelines developed for adults, whether in a hospital or out of a hospital setting, should be referenced. In the UK, the NEWS2 score is used for screening. In the context of this study, all patients identified through this process will be categorized as potential sepsis cases (i.e., severe infection). If there is confirmation based on the sepsis-3 definitions, the diagnosis of sepsis will be accepted.
For centers using the SIRS criteria for sepsis screening, at least two of the criteria must be met. When this criterion is fulfilled, these patients will also be classified as potential sepsis cases (i.e., severe infection). If the sepsis-3 definitions confirm the diagnosis, sepsis will be acknowledged.
Based on the proposed approach the following categories of infection severity will be applied:

- **Local:** findings of infection in absence of any systemic manifestations
- **Severe:** findings of infection in presence of systemic manifestations
- **Sepsis:** findings of infection in presence of systemic manifestations and organ failure

Table S2. Summary of criteria employed in classifying infection severity.

	SIRS or NEWS2	Organ failure
Local	No	No
Severe	Yes	No
Sepsis	Yes	Yes

Local infections include Superficial (SSI-S), deep (SSI-D), organ/ space (SSI-O), UTI and CAUTI causing local infection: cystitis, prostatitis, epididymitis, orchitis, moderate pyelonephritis. HAI- CDIF can also be classified as a local infection in absence of systemic findings, which is rare.

Organ failure will be identified using the SOFA criteria as shown in Table S3.

Table S3. SOFA score criteria

Parameter	Score: 0	Score: 1	Score: 2	Score: 3	Score: 4
PaO2/FIO2	< 400 mm Hg (53.3 kPa)	< 400 mm Hg (53.3 kPa)	< 300 mm Hg (40 kPa)	< 200 mm Hg (26.7 kPa) with respiratory support	< 100 mm Hg (13.3 kPa) with respiratory support
Platelets	≥ 150 × 10^3/mcL (≥ 150 × 10^9/L)	< 150 × 10^3/mcL (<150 × 10^9/L)	< 100 × 10^3/mcL (< 100 ×10^9/L)	< 50 × 10^3/mcL (< 50 × 10^9/L)	< 20 × 10^3/mcL (< 20 × 10^9/L)
Bilirubin	< 1.2 mg/dL (20 micromole/L)	1.2–1.9 mg/dL (20–32 micromole/L)	2.0–5.9 mg/dL (33–101 micromole/L)	6.0–11.9 mg/dL (102–204 micromole/L)	> 12.0 mg/dL (204 micromole/L)
Cardiovascular	MAP ≥ 70 mm Hg	MAP < 70 mm Hg	Dopamine < 5 mcg/kg/minute for ≥ 1 hour *or* Any dose of dobutamine	Dopamine 5.1–15 mcg/kg/minute for ≥ 1 hour *or* Epinephrine ≤ 0.1 mcg/kg/minute for ≥ 1 hour *or* Norepinephrine ≤ 0.1 mcg/kg/minute for ≥ 1 hour	Dopamine > 15 mcg/kg/minute for ≥ 1 hour *or* Epinephrine > 0.1 mcg/kg/minute for ≥ 1 hour *or* Norepinephrine > 0.1 mcg/kg/minute for ≥ 1 hour
Glasgow Coma Scale score*	15 points	13–14 points	10–12 points	6–9 points	< 6 points
Creatinine	< 1.2 mg/dL (110 micromole/L)	1.2-1.9 mg/dL (110–170 micromole/L)	2.0-3.4 mg/dL (171–299 micromole/L)	3.5-4.9 mg/dL (300–400 micromole/L)	> 5.0 mg/dL (440 micromole/L)

Urine output	—	—	—	< 500 mL/day	< 200 mL/day

Sepsis: Life-threatening organ dysfunction caused by a dysregulated host response to infection. For clinical application, organ dysfunction can be represented by an increase in the Sequential (Sepsis-related) Organ Failure Assessment (SOFA) score of 2 points or more

Septic shock: A subset of sepsis in which particularly profound circulatory, cellular and metabolic abnormalities are associated with a greater risk of mortality than with sepsis alone. Patients with septic shock can be clinically identified by a vasopressor requirement to maintain a mean arterial pressure of 65 mmHg or greater and serum lactate level greater than 2 mmol/l (>18 mg/dL) in the absence of hypovolemia.

Appendix S4 Data Collection Forms

There will be three forms that local investigators will need to fill in, which are:

- Hospital form data library: filled in once
- Patient screening form: short questionnaire to check patient eligibility. It will be registered in study logbook.
- Patient form: data collection form for eligible patients.

These Data Collection forms exist for DEEP-Uro RA/LP and are available upon request.

Appendix S5 Sample Size Calculation

Table S4 represents the assumptions underlying the study design and sample size calculation based on our prior systematic review of infectious complications following robot-assisted laparoscopic prostatectomy (RALP) (4). Sample size calculations were performed to estimate both primary and secondary indicators with predefined precision. For the first planned study focusing on RALP, the primary indicator assumes a 30-day postoperative infection rate of 5%. Based on this assumption, a sample size of 1825 patients is required to achieve a 95% confidence interval (CI) half-width of ±1.0%. For sepsis, assuming an incidence of 0.5%, a sample of 2,124 patients is required to reach a 95% CI precision of ±0.30%.

The planned enrolment of 2070 consecutive procedures is expected to yield precisions of approximately ±0.94% for postoperative infection and ±0.30% for sepsis. This level of precision is considered sufficient for audit purposes and allows for potential clustering effects across participating centers.

Table S4. Sample size calculation and precision for DEEP-URO RA/LP study

Outcome	Assumed incidence	Required Sample Size	95% CI Half-Width	Planned Enrolment Precision
30-day postoperative infection	5%	1825	±1.0%	±0.94%
Sepsis	0.5%	2124	±0.30%	±0.30%

CI=confidence interval; RA/LP= robot assisted and conventional laparoscopic radical prostatectomy
